# Comparative Bioavailability of Different Coenzyme Q10 Formulations in Healthy Elderly Individuals

**DOI:** 10.3390/nu12030784

**Published:** 2020-03-16

**Authors:** Igor Pravst, Juan Carlos Rodríguez Aguilera, Ana Belen Cortes Rodriguez, Janja Jazbar, Igor Locatelli, Hristo Hristov, Katja Žmitek

**Affiliations:** 1 Nutrition Institute, Tržaška cesta 40, SI-1000 Ljubljana, Slovenia; hristo.hristov@nutris.org (H.H.); katja.zmitek@vist.si (K.Ž.); 2 Biotechnical Faculty, University of Ljubljana, Jamnikarjeva 101, 1000 Ljubljana, Slovenia; 3 Department of Physiology, Anatomy and Cell Biology, Pablo de Olavide University, 41013 Seville, Spain; jcrodagu@upo.es (J.C.R.A.); abcorrod@upo.es (A.B.C.R.); 4 Faculty of Pharmacy, University of Ljubljana, Aškerčeva cesta 7, 1000 Ljubljana, Slovenia; janja.jazbar@ffa.uni-lj.si (J.J.); igor.locatelli@ffa.uni-lj.si (I.L.); 5 VIST—Higher School of Applied Sciences, Gerbičeva cesta 51A, SI-1000 Ljubljana, Slovenia

**Keywords:** coenzyme Q10, CoQ10, Q10Vital^®^, ubiquinone, ubiquinol, bioavailability, water-soluble formulations, the elderly

## Abstract

Coenzyme Q10 (CoQ10) plays a central role in mitochondrial oxidative phosphorylation. Several studies have shown the beneficial effects of dietary CoQ10 supplementation, particularly in relation to cardiovascular health. CoQ10 biosynthesis decreases in the elderly, and consequently, the beneficial effects of dietary supplementation in this population are of greater significance. However, most pharmacokinetic studies have been conducted on younger populations. The aim of this study was to investigate the single-dose bioavailability of different formulations of CoQ10 in a healthy geriatric population. A randomized, three-period, crossover bioavailability study was conducted on 21 healthy older adults (aged 65–74). The treatment was a single dose with a one-week washout period. Three different formulations containing the equivalent of 100 mg of CoQ10 were used: Q10Vital^®^ water-soluble CoQ10 syrup (the investigational product—IP); ubiquinol capsules (the comparative product—CP); and ubiquinone capsules (the standard product—SP). Ubiquinone/ubiquinol was followed in the plasma for 48 h. An analysis of the ratio of the area under the baseline-corrected concentration curve (ΔAUC_48_) for total CoQ10 and a comparison to SP yielded the following: The bioavailability of CoQ10 in the IP was 2.4-fold higher (95% CI: 1.3–4.5; *p* = 0.002), while the bioavailability of ubiquinol (CP) was not significantly increased (1.7-fold; 95% CI: 0.9–3.1, *p* = 0.129). No differences in the redox status of the absorbed coenzyme Q10 were observed between formulations, showing that CoQ10 appeared in the blood mostly as ubiquinol, even if consumed as ubiquinone.

## 1. Introduction

Coenzyme Q10 (CoQ10) is a highly lipophilic molecule that is naturally present in all membranes of human cells [1], where it plays a central role in the oxidative phosphorylation process. It is a carrier of electrons from complexes I and II to complex III, supporting the conversion of energy from carbohydrates and fatty acids into the energy-rich adenosine triphosphate [2]. In the body, it is biosynthesized from mevalonate (one of its key precursors), but there is evidence that the efficiency of this biosynthesis progressively decreases with age [1]. A number of foods provide an exogenous source of CoQ10, but its usual daily presence in people’s diets in Western countries is only 3–5 mg [3]. Much higher amounts are available in over-the-counter supplements [4].

In addition to its role in energy conversion, a number of potential health benefits of CoQ10 supplementation have been reported [5], with the strongest evidence emerging in terms of the aging process [6] and cardiovascular health [7]. Although in recent decades most trials have been considerably limited by their low numbers of subjects, the results of the Q-SYMBIO randomized controlled trial demonstrated that CoQ10 supplementation led to a reduction in major adverse cardiovascular events in a population with chronic heart failure [8,9], contributing to interest in this compound.

The key to effective CoQ10 supplementation is the enhancement of its bioavailability. Coenzyme Q10 is a substance with relatively high molecular weight (Mr = 863)—it is also insoluble in water and has limited solubility in lipids, and therefore it is poorly absorbed in the gastrointestinal tract [10]. Its absorption and uptake pathways are similar to those of vitamin E—these start with emulsification and the formation of micelles with fatty food constituents, which is facilitated by secretions from the pancreas and bile in the small intestine [11]. The absorption efficiency is also dose-dependent [11]. Additionally, CoQ10 intake with a meal can notably improve its absorption [11,12].

The pharmacokinetic parameters of orally ingested CoQ10 were investigated using deuterium-labeled CoQ10 [13]. Slow absorption in the gastrointestinal tract, which had been previously hypothesized, was confirmed (with a T_max_ of about 6 h), and a second plasma CoQ10 peak could be observed around 24 h after oral ingestion [13]. This second peak could have been attributable to both enterohepatic recycling and redistribution from the liver back into circulation, primarily via LDL/VLDL fractions [10]. Several physiological factors influence the bioavailability of compounds after oral intake, e.g., sex, age, genetic phenotype, the health of the gastrointestinal tract, various health disorders, the administration route, and interactions with food. There have been several promising efforts to enhance the bioavailability of CoQ10 using formulations. These efforts have been focused on reductions in its particle size and the modulation of its water solubility/dispersibility, e.g., using complexation, solubilization, or reduction [3]. In a recent study, very promising results using modified-release preparations were also reported [14].

CoQ10 is widely used as a food supplement throughout the world. Various formulations and dosages are marketed and used, e.g., powder-based compressed tablets, chewable tablets, powder-filled hard-shell capsules, soft gels containing oil suspensions, and syrups (generally around 100 mg/day) [4]. CoQ10 has an excellent safety record [15], even with chronic exposure to 900 mg/day [16]. The lethal single-dose administration over 5 g/kg was determined in rats [17]. That said, regulatory risk assessments have not resulted in any hazards related to supplementation with CoQ10 [18].

As explained above, supplementation with CoQ10 is most relevant in older people; therefore, it is surprising that the elderly have not been represented in the many studies that have investigated the single-dose bioavailability of CoQ10 [10]. We have only identified one single-dose bioavailability study that has been conducted on people older than 60 years. Evans and coworkers conducted a study on adults older than 60 years [19], enrolling 10 healthy persons as determined by laboratory results, a medical history, and a physical examination; however, the use of nonacute medications was not considered to be an exclusion criterion. We have found no studies that have been conducted on people aged 65 years or older, which is usually the criterion for “elderly” [20]. The situation with multiple-dose interventions is not much better, with very limited data available for older adults [21].

The aim of this study was to investigate the single-dose bioavailability of three CoQ10 formulations in a healthy geriatric population. The study was conducted as a single-center, randomized, three-period, crossover intervention using a single dose of 100 mg of CoQ10 with the following formulations: Capsules with ubiquinone as a standard product and two tested products (capsules containing ubiquinol, and syrup containing Q10Vital^®^ water-soluble CoQ10). The primary goal of the single-dose study was to find the relative bioavailability between the tested and standard product expressed as a ratio of the AUC_48_ (the area under the plasma concentration curve from the time of administration to the last observation point at 48 h) to the total CoQ10 plasma concentration above the baseline value. An additional objective was to compare the ratio of the reduced CoQ10 level to the total CoQ10 plasma level (using the AUC_48_) after the ingestion of all three formulations.

## 2. Experimental Section

### 2.1. Tested CoQ10 Formulations

The study was conducted with three different formulations:

- The standard product (SP): ubiquinone capsules (NuU Nutrition, York, UK; containing 100 mg ubiquinone per capsule);
- The investigational product (IP): syrup with Q10Vital^®^ water-soluble form of CoQ10 (Quvital^®^ syrup, Valens Int. d.o.o., Sencur, Slovenia; containing 100 mg of ubiquinone per 5 mL);
- The comparative product (CP): capsules with reduced form of CoQ10 (NOW^®^ Ubiquinol, Bloomingdale, IL, USA; containing 100 mg ubiquinol per capsule).

We used a single dosage of one capsule (SP, CP) and 5 mL of syrup (IP).

### 2.2. Blood Sample Preparation and Analytical Procedures

Blood samples (2 mL) were drawn into blood collection tubes containing Anticoag. K3-EDTA and centrifuged at 1500 *g* for 15 min at 5 °C. The plasma was separated and transferred in aliquots into three separate vials (with a minimum of 200 µL of plasma in each). To ensure an oxygen-free vapor phase gas inside the vials, labeled opened vials were maintained on dry ice prior to plasma transfer. The vials were closed immediately after plasma transfer, kept frozen at −80 °C, and sent on dry ice to the laboratory, where they were stored at −80 °C until analysis. The quantification of ubiquinone and ubiquinol in plasma was conducted in a biomedical laboratory at the Pablo de Olavide University (Seville, Spain) in line with previously described protocols [22] that were modified as follows: 125 µL plasma was mixed with 450 µL of ice cold 2-propanol containing 10 ppm (*v*/*v*) 2-mercaptoethanol (to scavenge residual oxygen during the extraction of lipid-soluble CoQ10 and to precipitate proteins). Coenzyme Q8 (100 pmol) was used as an external standard to normalize CoQ10 recovery and to confirm the unaltered redox status due to residual 2-mercaptoethanol. The extracted samples were mixed by vortex for 30 s, left for 3 min on ice, and mixed again by vortex for 30 s. Aggregated proteins were pelleted down by centrifugation at full speed in a refrigerated microfuge for 5 min. One-hundred microliters of supernatant was injected for HPLC analyses. Reduced CoQ10 was separated from its oxidized form using a methanol–propanol gradient method and electrochemical detection (as reported in [22]). Chromatographic conditions: Isocratic elution using methanol/1 M ammonium acetate, pH 4.4 (98:2), at 1.4 mL/min for 30 min. Column: Thermo Hypersil Gold aQ 250 mm × 4.6 mm, 5 µm Cat#0783107N thermostatized at 32 °C. Detection was done using an ESA Coulochem III set at −700 mV in channel #1 and then at +500 mV in channel #2. The mobile phase conditioning guard cell was +500 mV.

### 2.3. Study Population

The study population included healthy elderly subjects recruited from the Ljubljana area of Slovenia (Central Slovenia). Considering the guidelines for studies on geriatric populations [16], elderly persons were considered to be those aged 65 years and above. Only healthy subjects were included (the absence of any prescribed medication for a month prior to the study and during the study). Additional inclusion criteria were signed written informed consent form (ICF), a body mass index (BMI) between 20 and 29 kg/m^2^, a willingness to avoid the consumption of any food supplements except for vitamin D and calcium for at least 2 weeks before and during the study, and an ability to consume dairy and cereal products (because tested formulations were ingested together with a standardized breakfast, including low-lactose yoghurt and bread). The exclusion criteria included having taken any prescribed medication within 2 weeks of the beginning of the study; the intake of any food supplements within 2 weeks of the beginning of the study (except for vitamin D and calcium); hypotension; any clinically significant history of serious digestive tract, liver, kidney, cardiovascular, or hematological disease or diabetes; gastrointestinal disorders or other serious acute or chronic diseases; known lactose or gluten intolerances or food allergies (a limitation in terms of the standardization of meals); inadequate veins (in the opinion of the investigator) or known contraindication for placement of a dedicated peripheral line for venous blood withdrawal; drug and/or alcohol abuse; using any form of nicotine or tobacco; mental incapacity that precluded adequate understanding or cooperation; or the participation in another investigational study or blood donation within 3 months of this study or during this study.

A study power calculation (power 0.9, alpha 0.05) was done using literature data for ΔAUC_48_ values [19]. We calculated that 19 subjects would be sufficient to detect a 2-fold difference between the standard and tested product, with the coefficient of variation among the ΔAUC_48_ values being 100%. To compensate for possible dropouts among the study population (elderly persons), we decided to enlarge the study sample to 21 subjects.

### 2.4. Screening Visit

The subjects responded to an invitation published on the website of clinical research organization (CRO) Vizera d.o.o., Ljubljana, Slovenia, and the Nutrition Institute, Ljubljana, Slovenia, and its Facebook page. Potential subjects registered their interest using an online form, self-reporting their sociodemographic data. Those who were expected to be in compliance with the above-described inclusion/exclusion criteria were invited to a screening visit. Screening visits were done up to 2 weeks prior to the first product administration. When a subject visited the site, the study and all of its procedures were explained to the subject. If the subject agreed to participate in the study, they signed the ICF and received a Screening Number. All screening visits were conducted by a medical doctor. All inclusion/exclusion criteria were checked to make sure the subject was eligible for the study. Once eligibility was confirmed, the following information was collected: Age, gender, and ethnic origin of the subject; vital signs (body temperature, heart rate, and respiratory rate); height (cm) and body weight (kg); relevant medical history; and concomitant medication. Special instructions were given to the subject (including instructions on the use of any food supplements except for vitamin D and calcium, which were prohibited for the duration of the study).

### 2.5. Single-dose Comparative Bioavailability Study

A single-dose comparative bioavailability study was conducted using a single-center, randomized, three-period, crossover medicinal experiment. Blinding was not possible because some products were capsules and others were syrups, and therefore the experiment was done as an open-label experiment. The study was divided into three 48-h treatment periods separated by a 1-week washout period. A single-dose study was therefore completed in three visits (Periods A, B, and C). Considering parallel design and that there were 21 subjects, each of the three preparations was tested by all 21 subjects.

Randomization was done on the first day during Visit 1. After the subject’s general health was checked (vital signs), subjects were randomly assigned a Subject Number that corresponded to one of the following sequences: 1 (IP-CP-SP), 2 (IP-SP-CP), 3 (CP-IP-SP), 4 (CP-SP-IP), 5 (SP-IP-CP), or 6 (SP-CP-IP). For example, group 1 (IP-CP-SP) received the IP during Visit 1 (Period A), the CP during Visit 2 (Period B), and the SP during Visit 3 (Period C).

During visits 1, 2, and 3, the subjects arrived at the center at lunchtime the day before the administration of the study product. A standardized lunch and dinner were served. The subjects’ general health was checked (vital signs). Following an overnight fast, a blood sample was taken the next morning, and a single dose of the study product containing 100 mg of CoQ10 (according to the randomization scheme) was administered orally together with a standardized light breakfast (a piece of bread and lactose-free yoghurt) and 200 mL of water. A standardized lunch and dinner were served after 5 and 11 h of product administration, respectively. The collection of blood samples was at *t* = 0 (baseline) and at 1, 2, 3, 4, 5, 6, 8, 12, 24, and 48 h after administration. To assure the feasibility of the study, the subjects were hosted at the testing center during the intervention. They arrived at the testing center on Tuesday evening (dinner), and the intervention started on Wednesday morning. The last blood collection was done on Friday morning, when the subjects were dismissed until the next visit. All 21 subjects completed the study.

The study protocol was approved by the Slovenian National Medical Ethics Committee (Ministry of Health, Republic of Slovenia), identification number KME 89/07/17 (Approval letter ID 0120-326/2017-5, date of approval: 18.7.2017) (ClinicalTrials.gov ID: NCT03284814). The study was performed in compliance with the requirements of the local authorities. All subjects signed written informed consent form (ICF) before conduction of the study.

### 2.6. Data Processing and Analyses

The data were processed and analyzed using IBM SPSS (IBM SPSS Statistics for Windows, Version 25.0. Armonk, NY, USA: IBM Corp., Released 2017), Stata Statistical Software, Release 15 (College Station, TX, USA, StataCorp LLC), and Microsoft Excel 2016 (Redmond, WA, USA).

The following pharmacokinetic parameters were calculated: The time to reach peak plasma concentration (t_max_), maximum total CoQ10 plasma concentration (c_max_), maximum total CoQ10 plasma concentration above the baseline value (Δc_max_), area under the total CoQ10 plasma concentration curve from the administration time to the last observation point (AUC_48_), and AUC_48_ above the baseline value (ΔAUC_48_). The AUC values were calculated according to the linear trapezoidal method. Baseline-corrected values (ΔAUC_48_, Δc_max_) were calculated by subtracting the baseline values from the measured plasma concentration values. A pharmacokinetic analysis was performed using Stata Statistical Software, Release 15 (College Station, TX, USA, StataCorp LLC).

The following statistical parameters were calculated for the total CoQ10 pharmacokinetic parameters: Mean, standard deviation (SD), median and range (min and max), geometrical mean, and coefficient of variation (CV in %). For statistical tests, a logarithmic transformation of the parameter values (e.g., plasma concentrations and AUC values) was performed. A comparison of the differences in baseline concentration of the total CoQ10 between the tested formulations and between periods (A, B, C) was performed using several pairwise paired samples *t*-tests. A comparison in pharmacokinetic parameters between the tested formulations was done using an ANOVA. Here, a univariate ANOVA (general linear model) with one of the subjects as a random factor was applied, with ΔAUC_48_ and Δc_max_ as dependent variables. For the baseline-corrected pharmacokinetic parameters, the product (IP, CP, and SP), the treatment period (A, B, and C), and their interaction were applied as fixed factors. A Bonferroni post hoc test was applied to the product factors, allowing for a comparison between all of the products (IP vs. SP, IP vs. CP, and CP vs. SP). Additionally, the ratio between the reduced and total CoQ10 was also compared using a paired samples *t*-test. The significance level was set at 0.05. Statistical analyses were performed using IBM SPSS Statistics for Windows, Version 25.0 (Armonk, NY, USA, IBM Corp.).

## 3. Results

A total of 21 subjects (17 females, 4 males) aged above 65 years with a body mass index between 20 and 29 kg/m^2^ were included in the study (Table 1). The study was conducted as planned without protocol amendments. All subjects completed the study.

### 3.1. Baseline CoQ10 Levels

Baseline concentrations of total CoQ10 are presented in Table 2 for all three preparations. Statistical tests showed no difference in baseline concentration between formulations (Table 2, paired samples *t*-test, *p* > 0.05).

The baseline concentrations of total CoQ10 for all three periods (A, B, and C) were also calculated. There was no accumulation of total CoQ10 between study periods, meaning that the washout period was sufficient (the mean (SD) was 1.20 (0.55), 1.30 (0.38), and 0.86 (0.28) for periods A, B, and C, respectively). The difference between period A and period B was not significant (paired samples *t*-test, *p* > 0.05), whereas the baseline values in period C were even lower (paired samples *t*-test, *p* = 0.021).

### 3.2. Total CoQ10 Pharmacokinetic Profile

**Figure 1:**
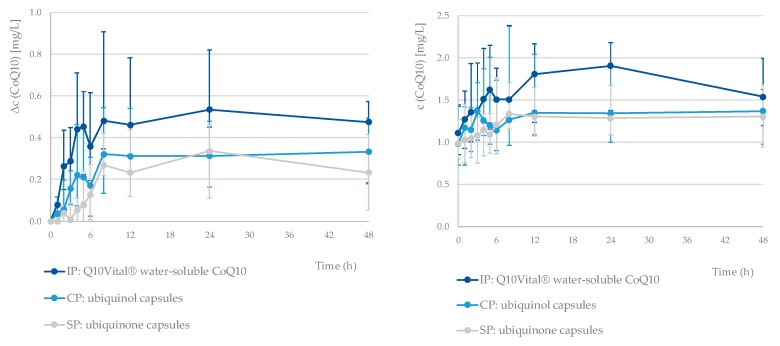
Median pharmacokinetic profile of Δ*c* and c (CoQ10, total) for each tested formulation. The error bars represent the first and third quartile of the plasma concentrations (*N* = 21 for each tested formulation). Standard product (SP) means ubiquinone capsules, comparative product (CP) means ubiquinol capsules, and investigational product (IP) means Q10Vital^®^ water-soluble CoQ10.

The median pharmacokinetic profiles for all three tested formulations are presented in Figure 1. The pharmacokinetic parameters for all three preparations are presented in Table 3. The mean t_max_ values observed were similar for all three tested formulations, but we should note that there was a relatively large SD. Comparison of t_max_ between investigational product vs. standard product as well as vs. comparative product for total CoQ10 showed no statistically significant differences (Wilcoxon Signed Ranks Test, *p* = 0.777 and *p* = 0.406, respectively). Notable differences were observed for both the Δc_max_ (geometrical mean (CV): 0.84 (56%), 0.62 (100%), and 0.41 (74%) mg/L for the IP, CP, and SP, respectively) and ΔAUC_48_ values (23.3 (56%), 15.9 (110%), and 9.5 (70%) mg/L for the IP, CP, and SP, respectively).

### 3.3. Comparison of ΔAUC_48_ for All Three Tested Formulations

The ΔAUC_48_ values were significantly different between products (ANOVA, *p* = 0.005). The results of the Bonferroni post hoc test showed a statistically significant difference between the investigational and standard products (*p* = 0.002, Table 4) and no difference between the investigational and comparative products (*p* = 0.366). We also did not observe a statistically significant difference between the comparative and standard products (*p* = 0.129). The effect of the treatment period (A, B, C) was also statistically significant (ANOVA, *p* = 0.037); however, the interaction between the treatment period and the products was not significant (ANOVA, *p* = 0.187), meaning that the effect of the treatment period did not influence the effect of the products on the ΔAUC_48_.

### 3.4. Comparison of Δc_max_ for All Three Tested Formulations

The Δc_max_ values were significantly different between products (ANOVA, *p* = 0.009). The results of the Bonferroni post hoc test indicated a statistically significant difference between the investigational and standard product (*p* = 0.004, Table 5) and no difference between the investigational and comparative product (*p* = 0.451). We also did not observe a statistically significant difference between the comparative and standard products (*p* = 0.167). The effect of the treatment period (A, B, and C) was also statistically significant (ANOVA, *p* = 0.014); however, the interaction between the treatment period and the products was not significant (ANOVA, *p* = 0.458), meaning that the effect of the treatment period did not influence the effect of the products on the Δc_max_.

### 3.5. Effect of the Intervention on the Plasma Redox Status of CoQ10

The results reported above corresponded with measurements of the plasma concentrations of total CoQ10, including both the reduced and oxidized forms. It should be noted that most of “total CoQ10” was attributable to its reduced form both during baseline measurements and after all three interventions (both with reduced and oxidized CoQ10). To enable comparisons between the tested formulations, we calculated the pharmacokinetic parameter ratios between the reduced and total CoQ10 (compared between the investigational product and standard product (Table 6) and between the investigational product and comparative product (Table 7)). We did not observe any significant differences between the tested formulations in terms of any of the parameters (paired samples *t*-test, *p* > 0.05): The ratio of the reduced vs. total CoQ10 was around 90% for all parameters.

## 4. Discussion

When orally administered, coenzyme Q10 is known to have limited bioavailability [11]. The substance has to pass the intestinal wall and then travel to the liver through portal circulation. An important limiting factor for absorption is insufficient time for absorption in the gastrointestinal tract. For instance, relatively high inter- and intraindividual variations can often be observed in cases of compounds with poor water solubility [23], as was the case in our study.

Different approaches have been used to improve the technological properties and bioavailability of CoQ10, enabling the development of new functional foods and pharmaceutical formulations [3]. Examples of such approaches are reductions in particle size (including the use of nanoparticles); the use of oil suspensions; the use of reduced CoQ10 (ubiquinol); and solubilization and increased water solubility, such as in the formation of molecular complexes [23]. In our study, we investigated the relative bioavailability of three different CoQ10 formulations using single doses (equivalent to 100 mg) of CoQ10 and a crossover approach. Two formulations with expected improved bioavailability (an inclusion complex of CoQ10 and β-cyclodextrin (Q10Vital^®^), and reduced CoQ10/ubiquinol) were compared to a standard formulation containing ubiquinone. The inclusion complex of CoQ10 and β-cyclodextrin was developed [24] with the intention of improving the water solubility, technological properties, and stability of CoQ10 [25], thereby enabling the preparation of water-based formulations, such as syrups. Improved bioavailability for this inclusion complex has been already shown in beagle dogs [26], and also in a human study [27], conducted using single doses (30 mg) in healthy individuals aged 30–52 years (39.6 ± 6.4 years). The improved bioavailability of ubiquinol has also been reported: in a single-dose study (100 mg) on individuals older than 60 years (67 ± 5 years) [19], and in a multiple-dose study on individuals over 55 years [21].

In our study, the intervention was conducted in conditions that were as controlled as possible. In contrast to most other bioavailability studies on CoQ10 [10], our subjects arrived at study center at lunchtime one day before each intervention. This enabled us to standardize lunch and dinner before the intervention. Following an overnight fast, the intervention started with the oral administration of the tested CoQ10 formulation, together with a standardized light breakfast and water. The after-intervention meals were also standardized. We believe that this approach was helpful in minimizing the effect of the subjects’ different dietary behaviors. We should note that the absorption efficiency of CoQ10 is also affected by several other parameters (including dose, if the administration is given with food, interactions with the food matrix, etc.) [11], so this limited possible comparisons to other studies.

The observed mean (SD) baseline total CoQ10 level in plasma in our study was 1.2 (0.6) mg/L, which is comparable to the baseline data reported by Zgang et al. (1.2 (0.4) mg/L in older adults above 55) [21] and Pozo-Cruz et al. (1.0 (0.42) mg/L in an elderly population) [28]. Interestingly, Evans et al. [19] have reported a much lower mean baseline total CoQ10 level in plasma among elderly participants (0.3 mg/L in subjects over 60), but it should be noted that they only recruited subjects with a plasma CoQ10 level between 0.2 and 1.0 mg/L. While originally, we also had the idea of using similar conditions in our inclusion criteria, we thought this would considerably lower our subject pool, which was already very limited due to the other inclusion/exclusion criteria (particularly age in relation to being healthy and the non-use of any medications). For example, 57% of our subjects (*N* = 12) had a baseline plasma CoQ10 level higher than 1.0 mg/L (week A data). It should also be noted that Pozo-Cruz et al. have reported a correlation between plasma CoQ10 levels and obesity in elderly persons, where obesity was related to lower CoQ10 levels [28]. However, as we enrolled only participants with BMIs up to 29 kg/m^2^, no such correlation was observed (Spearman’s ro = 0.245; *p* = 0.285) in this study. It should be noted that observed baseline plasma total CoQ10 levels were not lower than in studies, conducted on younger subjects [11,12].

A single-dose intervention with all three tested formulations resulted in a notable increase in plasma CoQ10 levels (Figure 1). A one-week washout period between the interventions was sufficient, as the statistical tests confirmed that there was no accumulation of CoQ10 between study periods. Interestingly, the lowest baseline CoQ10 concentration was observed before last treatment period (week C). Considering that study treatments have been carried out with three single dosages with relatively small amounts of CoQ10 over a longer period of time, it is very unlikely that this observation is related with a decrease in endogenous biosynthesis.

The bioavailability of the tested formulations was compared using the area under the baseline-corrected concentration curve (ΔAUC_48_). The lowest ΔAUC_48_ was observed for standard CoQ10 (with a geometric mean (CV) of 9.5 (71%)), while significantly higher value was observed for the investigational product (water-soluble Q10Vital^®^; 23.3 (56%)). On the other hand, ΔAUC_48_ of the comparative product (15.9 (110%); ubiquinol) was not significantly different in comparison with standard product.

We should note that the high standard deviations and differences in the individual pharmacokinetic profiles (Supplementary Figures S1–S3) indicated considerable differences between the subjects, but very similar relative standard deviations have also been observed in comparable studies [19]. A pairwise comparison showed that the relative bioavailability of CoQ10 in the investigational water-soluble Q10Vital^®^ (IP vs. SP ratio: 2.44; 95% CI: 1.33–4.50; *p* = 0.002) was higher, while in case of the comparative ubiquinol product, the difference was not statistically significant (CP vs. SP ratio: 1.66; 95% CI: 0.90–3.06; *p* = 0.129). We also did not observe statistically significant differences in the relative bioavailability between the investigational water-soluble Q10Vital^®^ and the comparative ubiquinol product (*p* = 0.366). Interestingly, in a single-dose (100 mg) study on older participants (>60 years), Evans et al. [19] reported more distinct differences between the bioavailabilities of ubiquinol and ubiquinone formulations; however, they did not test water-soluble CoQ10 formulations. On the other hand, López-Lluch et al. have recently reported the results of their single-dose study (100 mg CoQ10, several different formulations) on healthy young participants (18–33 years), where a ubiquinone formulation was notably more bioavailable than ubiquinol [29].

In our study, the mean maximum plasma CoQ10 concentration (Δc_max_) for the ubiquinol formulation was 0.90 mg/L, which is comparable to that reported by Evans et al. [19]. We observed a similar Δc_max_ for the investigational water-soluble Q10Vital^®^ (0.97 mg/L) and a notably lower value for standard ubiquinone (0.52 mg/L). Interestingly, in younger adults, López-Lluch et al. have reported a notably lower Δc_max_ in the case of ubiquinol formulation (0.49 mg/L), while they also observed the highest Δc_max_ in a formulation containing ubiquinone (0.95 mg/L) [29]. A similar range for Δc_max_ has also been reported in other single-dose studies using 100 mg of CoQ10 [11,29].

To gain further insight into this issue, we also investigated the redox distribution of the CoQ10 in the plasma after intervention with all three formulations. It has previously been suggested that either during or following absorption in the intestine, CoQ10 is reduced, resulting in an increase in the plasma ubiquinol level [11], and that the majority of the CoQ10 in circulation is in reduced form [30]. The pharmacokinetic parameter ratios between reduced and total CoQ10 in our study showed that this was also the case in elderly human subjects: The redox state of the CoQ10 in the tested formulations did not affect the CoQ10 redox state in plasma. For example, the proportion of ubiquinol in plasma was around 90% when participants consumed a formulation with ubiquinol or ubiquinone (Table 6 and Table 7).

A major strength of this study was that the intervention was done in a healthy adult population using a controlled crossover design, as this enabled us to avoid the effects of between-group differences, which can occur in parallel study designs. However, quite large interindividual differences were still observed in the baseline plasma CoQ10 levels (baseline CoQ10 range was not used as part of the inclusion/exclusion criteria). The inclusion of subjects with low baseline plasma CoQ10 level intervals would also have been beneficial in terms of lowering the observed deviation in this pharmacokinetic parameter, but such an approach would have made the study results less relevant for the general elderly population. It should also be noted that at our last measuring point (48 h) plasma CoQ10 concentrations were still above baseline for all three tested formulations. While many previous single-dose bioavailability studies have used an even shorter observation time [10], our results show that at least in an elderly population, a longer observation time would be beneficial. However, considering that in our case, the subjects were institutionalized for the entire observation time, this would considerably increase the burden for study participants. The participants arrived at our study center one day prior to the intervention, enabling us to have better control over the study conditions; however, this also extended the subjects’ institutionalization by one day per treatment, or three days per subject. Another limitation should be mentioned. Drug absorption rate can be affected by the degradation rate of pharmaceutical formulation. While this is not a problem in syrups, typical degradation time of pharmaceutical capsules in gastric juice is also within 5–10 min. Considering this, we did not conduct any dissolution tests. However, given the study observation time (48 h), we believe that in our case, the degradation rate of the pharmaceutical formulation did not notably affect the study results.

## 5. Conclusions

We observed considerable differences in the bioavailability of different CoQ10 formulations in an elderly population, where supplementation with this bioactive constituent is most relevant. We calculated the ratio of the area under the baseline-corrected concentration curve (ΔAUC_48_) for total CoQ10: The bioavailability of the investigational water-soluble Q10Vital^®^ syrup was 2.4-fold higher in comparison to standard ubiquinone capsules, while the difference in the bioavailability of comparative ubiquinol capsules was not statistically significant. It is important to also highlight that we did not observe significant differences between formulations in the redox status of the absorbed CoQ10, which indicates that CoQ10 appears in blood almost exclusively as ubiquinol, even if consumed as ubiquinone.

## Figures and Tables

**Table 1:** Study population demographic data (*N* = 21). BMI: Body mass index.

	Age (years)	Weight (kg)	Height (cm)	BMI (kg/m^2^)
Mean (SD)	68.5 (2.7)	68.4 (8.9)	167 (6.5)	24.7 (2.1)
Median (min/max)	68 (65/73)	70 (57/83)	166 (158/181)	25 (20/29)

**Table 2:** Baseline concentrations of total coenzyme Q10 (CoQ10) (in mg/L) for all tested formulations.

Formulation	Mean ^#^	SD	CV (%)	Median	Min	Max	*N*
IP	1.15	0.41	35.3	1.11	0.37	2.01	21
CP	1.07	0.42	39.3	0.98	0.52	1.89	21
SP	1.14	0.54	47.4	0.98	0.35	2.50	21

Standard product (SP): ubiquinone capsules; Comparative product (CP): ubiquinol capsules; Investigational product (IP): Q10Vital^®^ water-soluble CoQ10; CV: coefficient of variation. ^#^ No statistically significant difference in the baseline concentration of CoQ10 (paired samples *t*-test, *p* > 0.05).

**Table 3:** Pharmacokinetic parameters based on the measured and baseline-corrected total CoQ10 plasma concentrations for each product.

	Mean	SD	Median	Min	Max	Geo. Mean	CV (%)	*N*
**Investigational product (IP): Q10Vital^®^ water-soluble CoQ10**								
AUC_48_ (h∙mg/L)	82.3	26.0	89.1	32.9	125.0	78.0	31.6	21
ΔAUC_48_ (h∙mg/L)	27.0	15.1	22.7	8.2	63.2	23.3	55.7	21
c_max_ (mg/L)	2.13	0.71	2.09	1.04	3.64	2.01	33.3	21
Δc_max_ (mg/L)	0.97	0.55	0.82	0.22	2.24	0.84	56.2	21
t_max_ (h)	15.3	11.0	12	3	48			21
**Comparative product (CP): ubiquinol capsules**								
AUC_48_ (h∙mg/L)	75.3	38.7	63.8	32.8	178.8	67.4	51.3	21
ΔAUC_48_ (h∙mg/L)	23.9	26.2	13.4	3.2	109.6	15.9	109.7	21
c_max_ (mg/L)	1.97	1.14	1.55	0.78	5.05	1.72	58.0	21
Δc_max_ (mg/L)	0.90	0.90	0.50	0.20	3.61	0.62	100.1	21
t_max_ (h)	17.0	15.1	12	2	48			21
**Standard product (SP): ubiquinone capsules**								
AUC_48_ (h∙mg/L)	67.4	28.6	59.8	21.2	136.4	61.8	42.5	21
ΔAUC_48_ (h∙mg/L)	13.4	9.4	13.0	1.1	29.8	9.5	70.5	21
c_max_ (mg/L)	1.65	0.66	1.52	0.60	3.27	1.53	39.8	21
Δc_max_ (mg/L)	0.52	0.38	0.42	0.11	1.68	0.41	74.2	21
t_max_ (h)	17.0	15.1	12	2	48			21

AUC_48_: the area under the plasma concentration curve from the time of administration to the last observation point, i.e., at 48 h.

**Table 4:** Comparison of the ΔAUC_48_ values of the tested formulations.

	*N*	ΔAUC_48_ Ratio ^#^	Lower Value of 95% CI	Upper Value of 95% CI	*p*-Value *
IP vs. SP	21	2.44	1.33	4.50	0.002
IP vs. CP	21	1.47	0.80	2.70	0.366
CP vs. SP	21	1.66	0.90	3.06	0.129

^#^ Based on geometrical means; * Bonferroni post hoc test; Standard product (SP): ubiquinone capsules; Comparative product (CP): ubiquinol capsules; Investigational product (IP): Q10Vital^®^ water-soluble CoQ10.

**Table 5:** Comparison of the Δc_max_ values of the tested formulations.

	*N*	Δc_max_ Ratio ^#^	Lower Value of 95% CI	Upper Value of 95% CI	*p*-Value *
IP vs. SP	21	2.04	1.21	3.42	0.004
IP vs. CP	21	1.35	0.81	2.27	0.451
CP vs. SP	21	1.50	0.90	2.53	0.167

^#^ Based on geometrical means; * Bonferroni post hoc test; Standard product (SP): ubiquinone capsules; Comparative product (CP): ubiquinol capsules; Investigational product (IP): Q10Vital^®^ water-soluble CoQ10.

**Table 6:** Pharmacokinetic parameter ratios between reduced and total CoQ10, compared between the investigational product and standard product.

	*N*	Reduced/Total Geo. Mean for IP	Reduced/Total Geo. Mean for SP	IP/SP Geo. Mean (95% CI)	*p*-Value *
ΔAUC_48_ ratio	21	0.900	0.837	1.08 (0.89–1.29)	0.424
Δc_max_ ratio	21	0.875	0.848	1.03 (0.87–1.23)	0.711

* paired samples *t*-test on logarithmically (LN) transformed values; Standard product (SP): ubiquinone capsules; Investigational product (IP): Q10Vital^®^ water-soluble CoQ10.

**Table 7:** Pharmacokinetic parameter ratios between reduced and total CoQ10, compared between the investigational product and comparative product.

	*N*	Reduced/Total Geo. Mean for IP	Reduced/Total Geo. Mean for CP	IP/CP Geo. Mean (95% CI)	*p*-Value *
ΔAUC_48_ ratio	21	0.900	0.888	1.01 (0.88–1.17)	0.841
Δc_max_ ratio	21	0.875	0.965	0.91 (0.79–1.04)	0.148

* paired-samples *t*-test on logarithmically (LN) transformed values; Comparative product (CP): ubiquinol capsules; Investigational product (IP): Q10Vital^®^ water-soluble CoQ10.

## Supplementary Materials

The following are available online at https://www.mdpi.com/2072-6643/12/3/784/s1, Figure S1: Individual pharmacokinetic profiles for total CoQ10 for investigational product (IP; water-soluble Q10Vital^®^); Figure S2: Individual pharmacokinetic profiles for total CoQ10 for comparative product (CP; ubiquinol capsules); Figure S3: Individual pharmacokinetic profiles for total CoQ10 for standard product (SP; ubiquinone capsules).
