# Supplementary material for: Comparative Bioavailability of Different Coenzyme Q10 Formulations in Healthy Elderly Individuals
Source: Nutrients. 2020 Mar 16;12(3):784. doi: 10.3390/nu12030784 (PMC7146408; doi:10.3390/nu12030784)
Supplement: Supplementary file 1 [file nutrients-12-00784-s001.pdf]

Comparative Bioavailability of Different Coenzyme Q10 Formulations in Healthy Elderly Individuals

Supplementary Materials

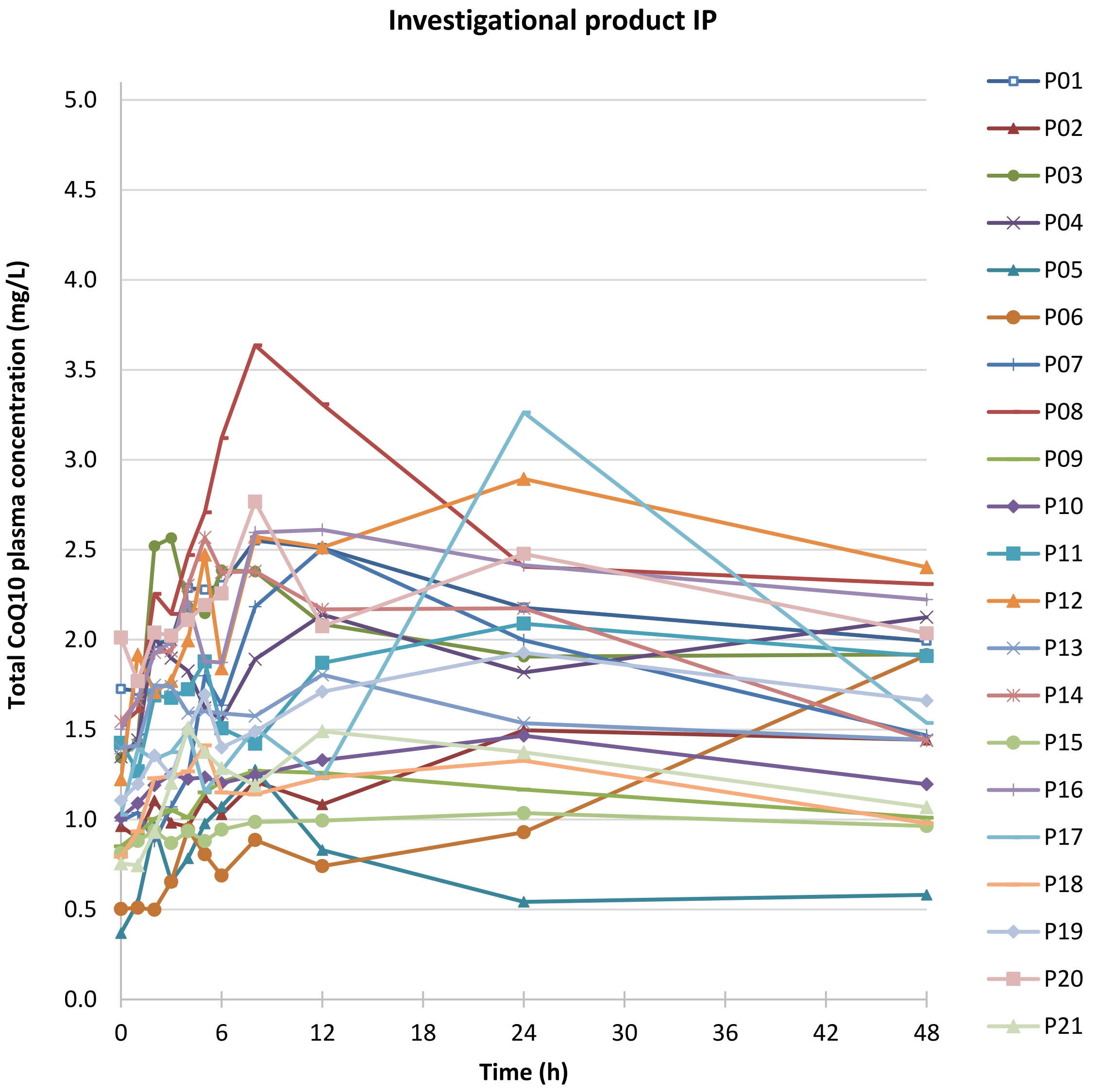

Figure S1. Individual pharmacokinetic profiles for total CoQ10 for investigational product (IP; water-soluble Q10Vital®).

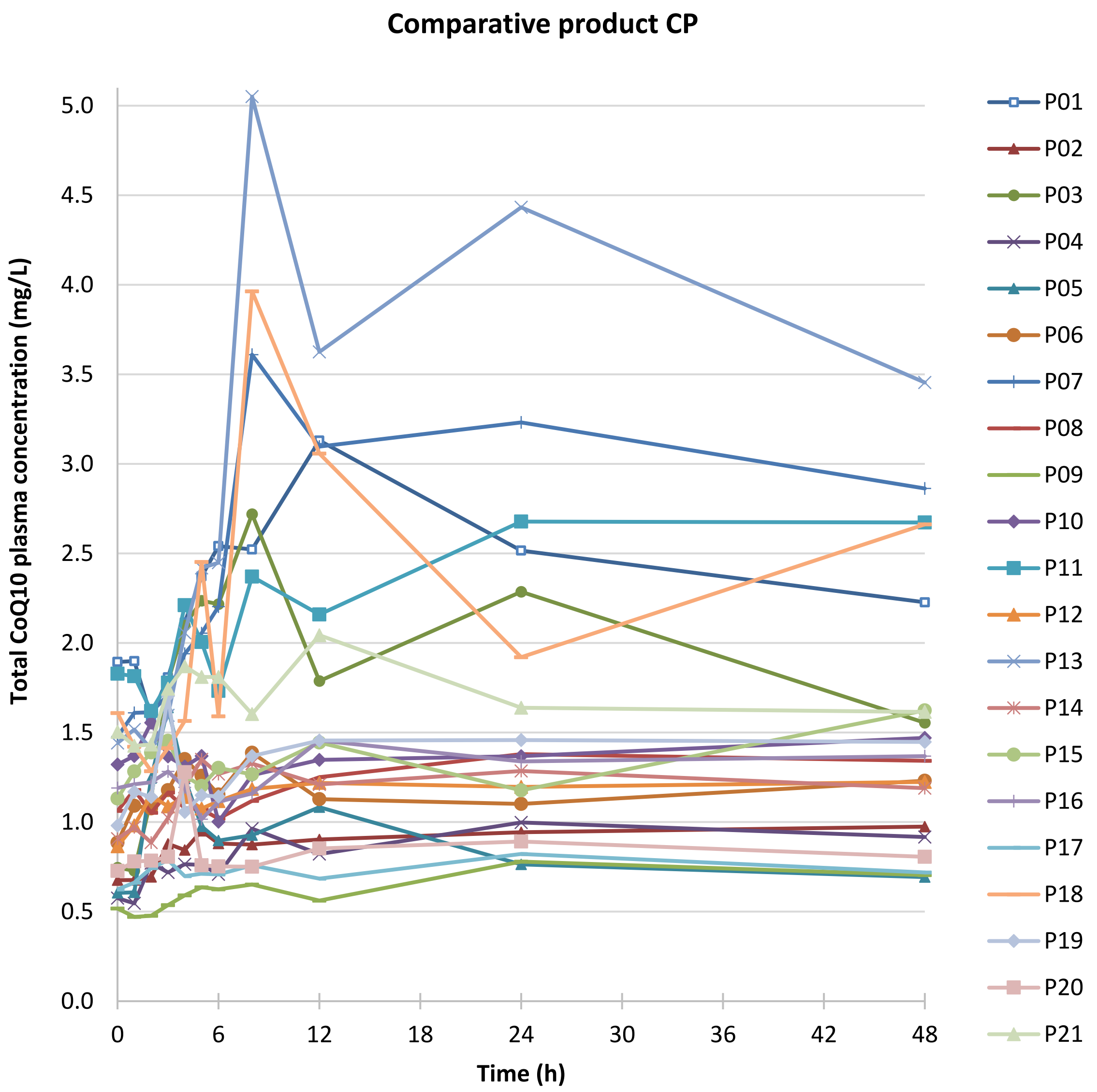

Figure S2. Individual pharmacokinetic profiles for total CoQ10 for comparative product (CP; ubiquinol capsules).

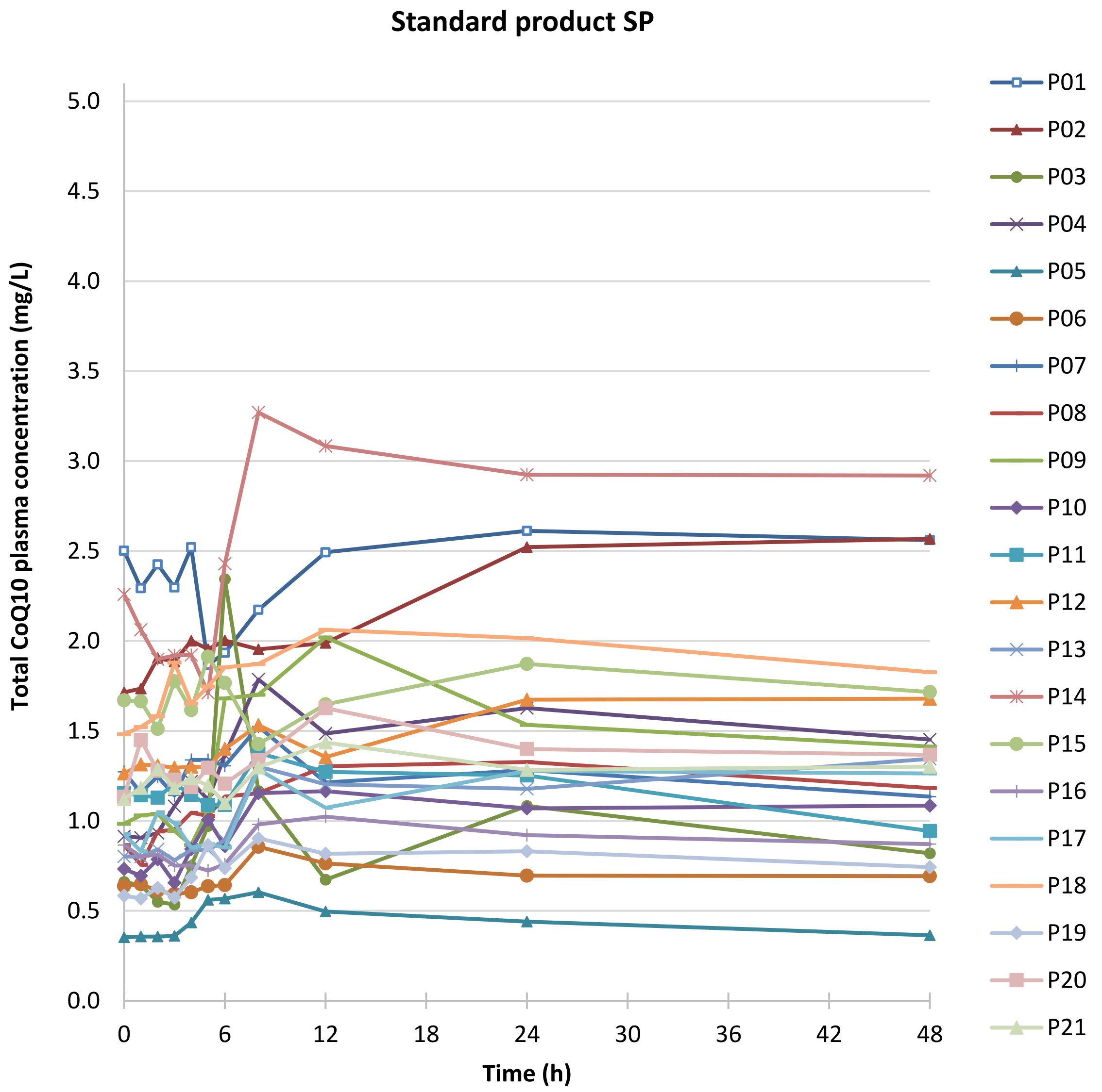

Figure S3. Individual pharmacokinetic profiles for total CoQ10 for standard product (SP; ubiquinone capsules).
